# Uncertainty Analysis of Drug Concentration in Pharmaceutical Mixtures

**Published:** 2008-06-03

**Authors:** Michalakis Savva

**Affiliations:** Division of Pharmaceutical Sciences, Arnold & Marie Schwartz College of Pharmacy and Health Sciences, Long Island University, Brooklyn, NY 11201

**Keywords:** uncertainty propagation analysis, powder mixtures, dosage forms, drug compounding

## Abstract

Using a Taylor expansion to first order, a novel method was developed to calculate the uncertainty of drug concentration in pharmaceutical dosage forms. The method allows, in principle, calculation of the maximum potential error in drug concentration in a mixture composed of an infinite number of ingredients that are measured on multiple balances of variable sensitivity requirements.

## Introduction

Unlike mathematics which is based on numbers-and numbers are exact- science is based on measurements. Measurements are composed of a number, a unit and an uncertainty that denotes the deviation of the measurement from the true or ideal value. Advances made in basic and applied sciences are intimately related to the level of accuracy and precision with which experiments are carried out and to the accuracy with which the levels of confidence associated with the measured quantities are estimated.

Quite frequently experimentally measured quantities have to be combined in some way in order to determine some other derived quantity. For example, in order to find the uncertainty in drug concentration of a drug that was measured on a balance of a particular sensitivity and mixed with excipients measured on a balance of same or different sensitivity, the uncertainties in the measured quantities have to be combined appropriately. Although the rules that govern uncertainty propagation calculations are well developed [1], they were never applied to carry out uncertainty analysis of ingredient concentration in mixtures. In this paper, the author uses differential calculus and develops a method that combines uncertainties in the measurements of drug and excipients to effectively calculate the uncertainty of drug concentration in pharmaceutical mixtures.

## The Method

The *key idea* in determining the uncertainty associated with drug concentration in drug-excipient mixtures is to produce a function that describes the variation of the drug quantity in the whole mixture, as shown below:

(1) $$f\;=\;\frac{x}{x\;+\;y\;+\;z\;+......}$$

*x* = drug quantity of interest

*y, z ….* = other ingredients

*f* = fraction or weight concentration of the drug in the total mixture.

*Equation* 1, is continuous in the domain of our interest since *x*, *y, z,*….. are real positive numbers. The potential error associated with *f* can be found from the total differential of the function above, as shown below:

(2) $$\begin{array}{l} f\;=\;f(x,y,z,\;....)\\ \;\;\;\;\;\;\;\;\;\;\;\;\;\;\;\;\;\;\;\;\;\;\;\;\;\;\Rightarrow \;df\;=\;{\left|\frac{\partial f}{\partial x}\right|}_{y,z,\ldots }\;dx\;+\;{\left|\frac{\partial f}{\partial y}\right|}_{x,z,\ldots }\\ \;\;\;\;\;\;\;\;dy+{\left|\frac{\partial f}{\partial z}\right|}_{x,y,\ldots }dz+\ldots \ldots \end{array}$$

*df, dx, dy, dz* are the uncertainties associated with the measurement of the corresponding quantities, *f, x, y* and *z*. More specifically, *dx, dy, dz* are equal to the sensitivity or readability of the balances utilized to measure the corresponding quantities, *x, y* and *z*. Performing the calculus of partial derivatives in *Equation* 1, yields:

(3) $$\begin{array}{l} \frac{df}{f}=\frac{y+z+\ldots }{x}\cdot \frac{dx}{\left(x+y+z+\ldots \right)}\\ \;\;+\frac{dy+dz+\ldots }{\left(x+y+z+\ldots \right)} \end{array}$$

$\frac{df}{f}$ is the relative uncertainty in *f*, i.e. drug concentration by weight in the mixture.

*Equation* 3, denotes the fractional or relative uncertainty of drug concentration in the mixture, that is,

(4) $$\frac{df}{f}=\left|\frac{\text{uncertainty}\;\text{in}\;f}{\text{ideal}\;\text{value}\;f}\right|$$

To better investigate the effect of the # of ingredients composing the mixture *n*, on the uncertainty of drug concentration, *Equation* 4 was expanded for fixed balance sensitivity.

Starting from *Equation* 3 and assuming use of same balance to measure all ingredients, i.e. *dx* = *dy* = *dz* = …

(5) $$\Rightarrow \frac{df}{f}=\frac{dx}{x}\cdot [1+(n-1)f]$$

*n* = total # of ingredients composing the mixture; drug *x* is excluded

A number of conclusions can be summarized from *Equation* 5:

1. $\frac{df}{f}=\frac{dx}{x}$ if the drug is mixed with a single ingredient (*n* = 1) and measured on the same balance. This result suggests that for a twocomponent mixture we only need to calculate the uncertainty associated with the drug measurement and we need not be concerned with the uncertainty associated with the measurement of the second ingredient present in the mixture.
2. The uncertainty in drug concentration in the mixture increases with the number of other ingredients assuming that *f*, *x* and *dx* are constant as shown in Figure 1a and b. Recognize, however, that both Figures indicate that the uncertainty in drug concentration is predominantly affected by the sensitivity of the balance.

The values for *x*, *dx*, and *f* were 0.5 g, 0.01 g and 0.0071 g, respectively for both figures. Both plots clearly indicate that as *n* → 1, $\frac{df}{f}\to \frac{dx}{x}=0.02$. These conclusions are valid for virtually all possible drug fractional masses, *f*, i.e. within the domain [1,0].

## Conclusions

Using a rational approach and differential calculus, a function was created and a 1st degree Taylor polynomial was derived, respectively, to calculate the uncertainty of drug quantity in a mixture. This novel method can, in principle, calculate the “maximum interval” uncertainty in drug concentration in a mixture that is composed of an infinite number of ingredients and the ingredients are measured using balances of same or different sensitivity. This “core” equation can be used to: (1) study the maximum interval uncertainty variation as a function of the number and corresponding masses of ingredients and the sensitivities of the balances used (2) determine a balance of appropriate sensitivity needed to measure drug concentrations within the range of a maximum allowable uncertainty and (3) to determine the least allowable weight of an ingredient within a maximum interval uncertainty.

## Figures and Tables

**Figure 1a.**
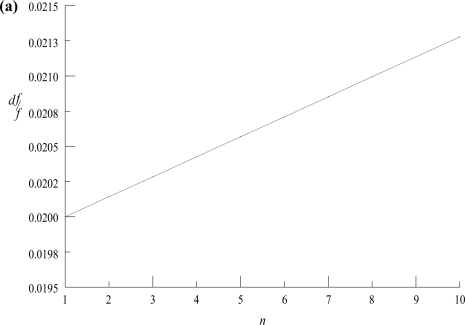
Relative uncertainty in drug concentration at constant drug quantity in the mixture utilizing a single balance for measurement of all ingredients, as a function of the total # of ingredients *n*, using *equation 5*. The slope of the line is equal to *dx/x.*

**Figure 1b.**
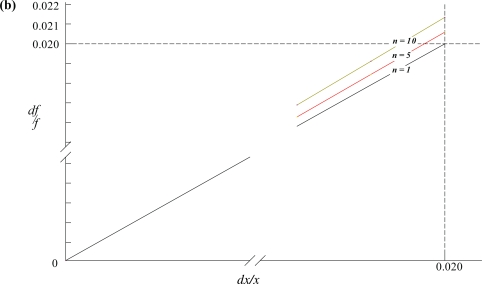
Contour lines of constant *n* using *equation 5*. The value of *n* for each contour is also shown. *Equation 5* indicates that the slope of the contour lines is given by [1+(*n*–1)*f*].
